# Biological Diagnosis of Ocular Toxoplasmosis: a Nine-Year Retrospective Observational Study

**DOI:** 10.1128/mSphere.00636-19

**Published:** 2019-09-25

**Authors:** Valentin Greigert, Alexander W. Pfaff, Arnaud Sauer, Denis Filisetti, Ermanno Candolfi, Odile Villard

**Affiliations:** a Institut de Parasitologie et Pathologie Tropicale, Université de Strasbourg, Strasbourg, France; b Laboratoire de Parasitologie et Mycologie Médicales, Hôpitaux Universitaires de Strasbourg, Strasbourg, France; c Centre National de Référence Toxoplasmose—Pôle sérologie, Hôpitaux Universitaires de Strasbourg, Strasbourg, France; d Service d’ophtalmologie, Hôpitaux Universitaires de Strasbourg, Strasbourg, France; University at Buffalo

**Keywords:** ocular toxoplasmosis, parasite, retinochoroiditis, diagnosis, ophthalmology, *Toxoplasma gondii*, diagnostics, infectious disease, ocular immunology, parasitology

## Abstract

Ocular toxoplasmosis (OT), i.e., the ocular manifestation of Toxoplasma gondii infection, is one of the leading causes of posterior uveitis. While ocular lesions are often typical, atypical forms often require biological confirmation of the diagnosis. Our study sought to review the biological OT diagnoses made in our laboratory to further assess the role of each test in the diagnostic procedure. All ocular samples sent to our laboratory over the last 9 years for OT diagnosis were included. These samples were analyzed using T. gondii PCR and antibody detection by means of immunoblotting and Candolfi coefficient (CC) determinations, either alone or in combination. Since serum analysis is required to interpret both the CC and immunoblotting, blood serology for T. gondii was also performed in most cases. Of the 249 samples analyzed, 80 (32.1%; 95% confidence interval [95%CI], 26.3 to 37.9) were positive for OT. Of these 80 cases, 52/80 (65.0%; 54.6 to 74.5) displayed a positive PCR, 15/80 (18.8%; 10.2 to 27.3) a positive CC, and 33/80 (41.3%; 95%CI, 30.5 to 52.0) a positive immunoblot result. Overall, 63 of the 80 OT diagnoses (78.8%; 95%CI, 69.8 to 87.7) were made on the basis of a single positive test result. Our study results remind us that current biological diagnostic tools for OT must be employed in combination to obtain an optimal diagnosis based on the precious ocular fluids sampled by ophthalmologists. Clinicobiological studies that are focused on correlating the performances of the different tests with clinical features are critically needed to improve our understanding of the pathophysiology and diagnosis of OT.

**IMPORTANCE** Ocular toxoplasmosis (OT), a parasitic infection of the eye, is considered to be the most important infectious cause of posterior uveitis worldwide. Its prevalence is particularly high in South America, where aggressive Toxoplasma gondii strains are responsible for more-severe presentations. The particular pathophysiology of this infection leads, from recurrence to recurrence, to potentially severe vision impairment. The diagnosis of this infection is usually exclusively based on the clinical examination. However, the symptoms may be misleading and are not always sufficient to confirm a diagnosis of OT. In such cases, biological tests performed by means of several techniques on blood and ocular samples may facilitate the diagnosis. In this study, we analyzed the tests that were performed in our laboratory over a 9-year period every time OT was suspected. Our report highlights that the quality of ocular sampling by ophthalmologists and combinations of several techniques are critical for a reliable biological OT diagnosis.

## Abstract (precis)

Ocular toxoplasmosis (OT), a parasitic infection of the eye, is considered to be the most important infectious cause of posterior uveitis worldwide. Its prevalence is particularly high in South America, where aggressive Toxoplasma gondii strains are responsible for more-severe presentations. The particular pathophysiology of this infection leads, from recurrence to recurrence, to potentially severe vision impairment. The diagnosis of this infection is usually exclusively based on the clinical examination. However, the symptoms may be misleading and are not always sufficient to confirm a diagnosis of OT. In such cases, biological tests performed by means of several techniques on blood and ocular samples may facilitate the diagnosis. In this study, we analyzed the tests that were performed in our laboratory over a 9-year period every time OT was suspected. Our report highlights that the quality of ocular sampling by ophthalmologists and combinations of several techniques are critical for a reliable biological OT diagnosis.

## INTRODUCTION

Ocular toxoplasmosis (OT) is the ocular presentation of the infection caused by the ubiquitous apicomplexan parasite Toxoplasma gondii. In some parts of the world, the prevalence of T. gondii infection is estimated at up to 80%, leading OT to represent one of the primary etiologies of posterior uveitis in regions like South America (1, 2). OT causes retinochoroiditis, leading to visual impairment from recurrence to recurrence and, in rare situations, to loss of sight in the infected eye (3, 4). In most cases, retinal lesions are sufficiently characteristic to allow an OT diagnosis to be established by ophthalmologists relying only on ophthalmic examination (5). However, the clinical presentation can at times prove to be misleading, requiring biological tests to be either confirmed or refuted (5–8). Indeed, a recent article showed that, in South America, the clinical diagnosis could be modified in a significant proportion of uveitis cases when adding laboratory testing (9). In these cases, physicians have resorted to tests performed on blood and ocular samples, these latter primarily consisting of aqueous humor (AH) samples collected through anterior chamber puncture (ACP), a fast and safe procedure (10, 11), but also of vitreous humor (VH) samples collected via vitrectomy, a far more risky intervention.

Several biological techniques enable clinical parasitologists to optimize these blood and ocular samples, including the direct detection of parasites through PCR and antibody detection with titer interpretation from the blood, as well as from ocular samples, as already reviewed elsewhere (12). By combining these techniques, clinical parasitologists are now able to achieve sensitivity (Se) and specificity (Sp) of up to 97% and 93%, respectively (13, 14). However, biological techniques, particularly PCR, might have different performances according to whether they are performed on European/North American or South American patient samples (15).

In the current study, we aimed to review all the OT cases diagnosed in our laboratory, without any consideration for the underlying clinical situation, so as to further assess the role of each test employed in positive samples according to the diagnostic procedure.

## RESULTS

From 1 January 2010 to 31 December 2018, 249 ocular samples primarily comprising AH (240/249) (96.4%; 95% confidence interval [95%CI], 94.1 to 98.7) were sent to our laboratory for OT diagnosis (see Table S1 in the supplemental material).

10.1128/mSphere.00636-19.1 TABLE S1 Results of the tests performed for OT diagnosis on the 249 samples analyzed in the Strasbourg University Hospital laboratory from 2010 to 2018. Download Table S1, PDF file, 0.05 MB. Copyright © 2019 Greigert et al. 2019 Greigert et al. This content is distributed under the terms of the Creative Commons Attribution 4.0 International license.

Blood serology analysis for diagnosis of toxoplasmosis was performed in our laboratory in 217 cases, of which 148/217 (68.2%; 95%CI, 62.0 to 74.4) were positive with a chronic infection profile, 7/217 (3.2%; 95%CI, 0.9 to 5.6) had a primary infection profile, and 1/217 was positive yet doubtful with respect to antibody avidity. PCR was performed in 242 cases, of which 52 (21.5%; 95%CI, 16.3 to 26.7) were positive. PCR was not performed in seven cases due to either insufficient AH quantity (*n* = 3) or for unknown reasons (*n* = 4). Ocular serology testing was conducted by means of immunoblotting in 117 cases, of which 51/117 (43.6%; 95%CI, 34.6 to 52.6) exhibited similar immune profiles in the blood and AH samples and 33/117 (28.2%; 95%CI, 20.1 to 36.4) different profiles, whereas 33/117 (28.2%; 95%CI, 20.1 to 36.4) were negative. Finally, the Candolfi coefficient (CC) was assessed in 83 cases, of which 15/83 (18.1%; 95%CI, 9.8 to 26.4) were positive, 26/83 (31.3%; 95%CI, 21.4 to 41.3) were negative, 32/83 (38.6%; 95%CI, 28.1 to 49.0) were noninterpretable due to blood-ocular barrier (BOB) permeability, and 10/83 (12.1%; 95%CI:5.0 to 19.0) were doubtful due to intermediate values. Only 74/249 (29.7%; 95%CI, 24.0 to 35.4) of all eye samples were tested using these three techniques.

**FIG 1:**
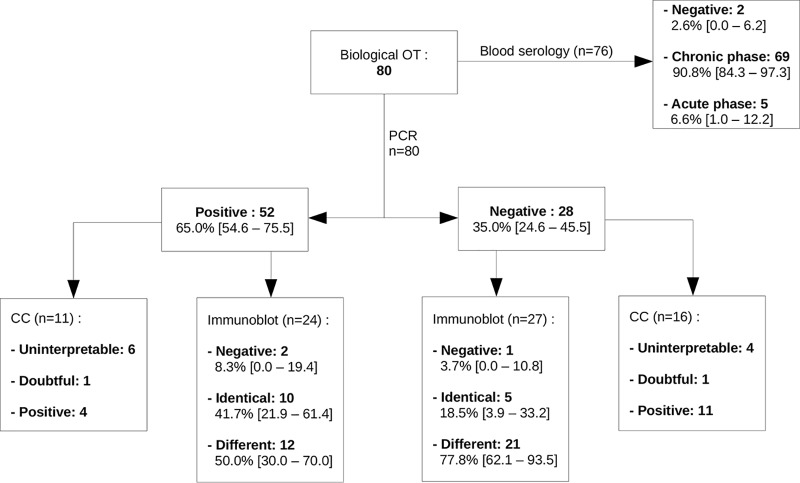
Results of biological tests in all OT biologically diagnosed at Strasbourg University Hospital from 1 January 2010 to 31 December 2018.

**FIG 2:**
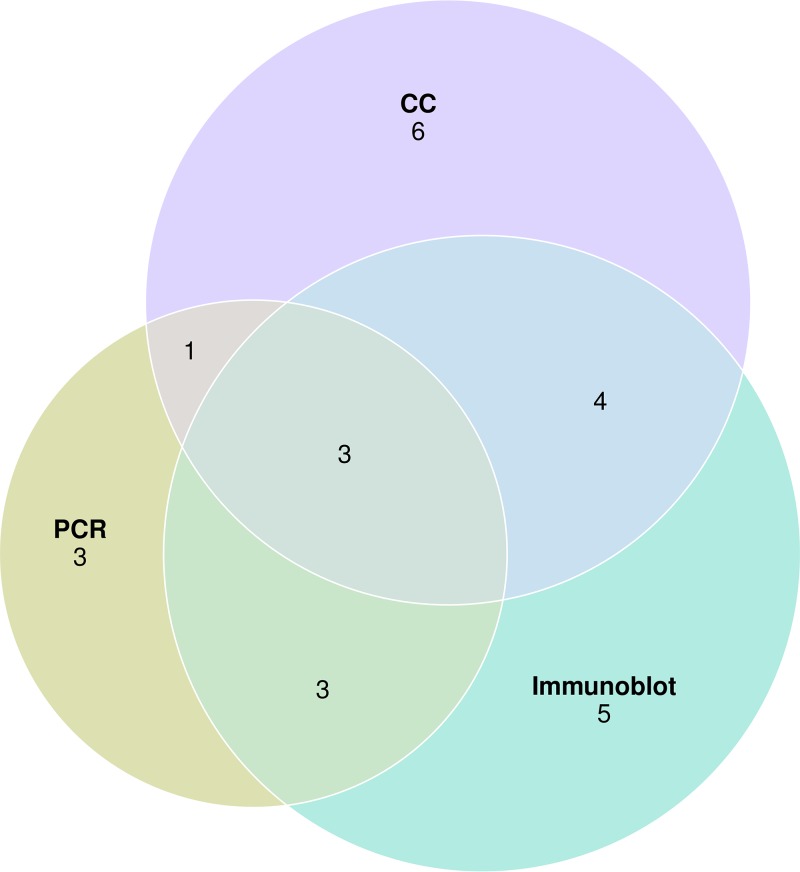
Euler diagram showing the number of positive tests for each ocular toxoplasmosis sample tested using PCR, immunoblotting, and Candolfi coefficient.

Of the 249 samples analyzed, 80 (32.1%; 95%CI, 26.3 to 37.9) produced results in favor of the OT diagnosis (Fig. 1). The PCR was positive in 52/80 cases (65.0%; 95%CI, 54.6 to 74.5); immunoblotting was positive in 33/80 cases (41.3%; 95%CI, 30.5 to 52.0), and the CC was positive in 15/80 cases (18.8%; 95%CI, 10.2 to 27.3). Blood serology was performed in our laboratory in 76/80 of positive cases, of which 74/76 turned out to be positive (97.4%; 95%CI, 93.8 to 100.0), with a primary infection profile in 5/76 cases (6.6%; 95%CI, 1.0 to 12.2). Of the two patients with negative blood serology, one displayed a positive yet noninterpretable CC due to a permeable BOB, whereas the other one did not undergo the ocular serologic assays. Of note, two of the five cases with a primary infection profile had a negative PCR. Of the 52 cases with a positive PCR, 25 were tested using either immunoblotting or CC, with 13 being positive (48.0%; 95%CI, 28.4 to 67.6); similarly, of the 41 cases with a positive CC or immunoblotting, 13 (31.7%; 95%CI, 17.5 to 46.0) were positive using PCR. Overall, 21 of the PCR-negative samples exhibited immune profiles that differed between the blood and eye samples, allowing the OT diagnosis to be established. This was confirmed by a positive CC in four cases. A total of 11 samples were analyzed through CC despite a positive PCR; among the 11 samples, 4 were positive, 6 noninterpretable due to BOB permeability, and 1 doubtful because of intermediate values. Finally, five samples displayed an interpretable and positive CC, allowing the OT diagnosis to be made. Overall, 63/80 OT diagnoses (78.8%; 95%CI, 69.8 to 87.7) were made due to a single positive test, whereas only 25/80 (31.2%; 95%CI, 21.1 to 41.4) were tested using the three techniques, of which only 3/25 (12.0%; 95%CI, 0.0 to 24.7) turned out to be positive with the three tests (Fig. 2).

Of these 249 samples, 124 (49.8%; 95%CI, 43.6 to 56.0) were additionally tested for viral infections (cytomegalovirus [CMV], herpes simplex virus [HSV], or varicella-zoster virus [VZV]), among which 11 (8.9%; 95%CI, 3.9 to 13.9) were positive (6 for HSV, 4 for VZV, and 1 for CMV), while 3 revealed the presence of PCR inhibitors. All the samples that were positive for a viral infection were negative for toxoplasmosis. In addition, 18 (7.3%; 95%CI, 4.0 to 10.5) of the 249 samples were tested for the presence of bacteria using PCRs (for detection of 16S DNA, *Borrelia* sp., *Bartonella* sp., or Chlamydia trachomatis), though all turned out to be negative. Of the 249 samples, 134 (53.8%; IC9%: 47.6 to 60.0) were also tested for other infections. Of the 169 samples that were negative for OT, 108 (63.9%; 95%CI, 56.7 to 71.2) were tested for other microorganisms, either viral or bacterial in nature, resulting in 11 additional microbiological diagnoses. Of these 11 subjects, 5 exhibited negative blood T. gondii serology.

## DISCUSSION

We performed an observational study over a 9-year period, allowing us to analyze data regarding a great number of samples. However, this kind of study, without prior design, did not allow us to access reliable clinical data about the patients whose samples were analyzed, and we could not ascertain whether the same set of techniques was always used for all analyses of the samples.

Over this 9-year period, our laboratory biologically diagnosed 80 OT cases based on 249 samples analyzed. This relatively low ratio highlights the fact that OT diagnosis primarily relies on ophthalmic examination in most cases. Indeed, in cases where the lesions found are typical of OT, clinicians often avoid performing an ACP (5, 16). Considering that most samples analyzed in our laboratory originated from patients presenting lesions that were compatible with though not typical of a diagnosis of OT, this ratio would indicate that biological confirmation is an essential diagnostic step in atypical OT presentations. In addition, when suspecting other ocular diseases, it is highly probable that clinicians take advantage of the ACP that they perform to assess the possibility of an atypical OT presentation, even when the suspicion is weak (17). This is illustrated by the fact that more than half of the samples were also analyzed for other kinds of microorganisms, either viral or bacterial. That situation artificially decreases the ratio of confirmed biological OT diagnoses, since tests are performed in settings where OT is, in fact, not clinically suspected. Finally, we cannot exclude the possibility that this low ratio might reflect low sensitivity of our diagnosis algorithm.

Of these 80 cases, 52 exhibited a positive PCR. This ratio highlights the relevance of this particular test in our diagnosis algorithm, since two-thirds of our cases could have been diagnosed using a single test on ocular samples. In the present study, the PCR was positive in three patients with an immune profile compatible with a primary infection, as well as in two patients presenting a negative serology, corresponding to either immunocompromised subjects or to very early primary infections—two situations in which PCR is believed to be highly sensitive. However, the PCR results remained negative in two subjects presenting immune profiles compatible with primary infection. The biological diagnosis of these two patients was made because of the CC, since the immunoblotting profiles of the blood and eye samples were similar. This is consistent with the course of a primary infection acquired through *per os* contamination, that is, systemically. However, since the CC analyzes the differences in the intensities of immunoglobulin synthesis in the eye and blood, rather than differences in antibody repertoires, we were able to detect strong intraocular antibody synthesis corresponding to retinochoroiditis. In this assay, the “mumps” ratio is necessary to assess the impermeability of the BOB. We have chosen to use the parameter of mumps virus immunity since, as previously explained, a large proportion of the French population has become immune to this virus following several vaccination campaigns (18). The same objective could, however, be achieved using virtually any other common microorganism generating long-lasting immunity and not involved in ocular pathology.

The PCR was negative in 28/80 OT cases. OT was then diagnosed using techniques based on detection of different anti-T. gondii antibody expression results, either with respect to the repertoire or the amplitude, between the blood and intraocular fluids. This could reflect the different kinetics of detection of parasites and antibodies by the tests used, during OT. Indeed, antibodies are expected to be expressed in the eye after the onset of infection, even in cases of recurrence (19, 28). In immunocompetent patients at least, under conditions of immune response pressure, given that the parasite presence *in situ* is probably at a low level and transient, we would expect patients with a positive PCR result to exhibit low ratios of positive immunoblotting or CC and patients with a negative PCR result to exhibit high ratios. Indeed, less than half of the positive PCR samples tested using antibody detection-based tests were positive for the latter, and, similarly, less than a third of the positive CC or immunoblot samples were positive using PCR. Nevertheless, as our analysis relied on real-life data, a small proportion of the samples underwent all three assays, thereby decreasing our statistics’ power and allowing for study biases. It may also be possible that these figures highlight a lack of sensitivity in the tests that we performed. It is, however, likely that the combination of these three assays enables the clinical parasitologists to perform the best test for each step of toxoplasmic retinochoroiditis, resulting in higher overall diagnostic performances, as previously described (13, 14, 20).

In our study, 175/249 of all the ocular samples were not tested using all three techniques; among those 175 samples, 42 displayed a positive PCR. Of the remaining 133 samples with a negative PCR, 86 were not tested with either immunoblotting or CC, and 47 were tested using only one of these tests. Most often, because of insufficient sample volume, these tests were not performed. In regard to the very high specificity of PCR, choosing not to perform antibody detection-based assays on samples already positive by PCR appears understandable, but it remains true that 133/249 (53.4%; 95%CI, 47.2 to 59.6) were not tested with the best of current diagnosis techniques, the use of which might have facilitated the OT diagnosis. This further highlights the importance of ensuring the quality of ocular liquid sampling by ophthalmologists in order to obtain reliable biological test results.

Finally, the performances of the tests that we applied could be compared with the results of a previous study carried out in the same laboratory and published in 2003. For instance, analyzing only the ocular samples tested with all three techniques, we showed a current PCR sensitivity of 40% (10/25) versus 28% PCR sensitivity in the data from 2003 (20). Moreover, the sensitivities of immunoblotting and CC were 33% and 53% in our previous study versus 60% (15/25) and 56% (14/25) in the current study, respectively. At that time, the PCR technique differed in that it used primers targeting a sequence in the B1 gene, with a lower sensitivity (21–23). Thus, these current results may likewise highlight the improvements made in the PCR technique, resulting in an increase in this assay’s sensitivity. However, if the hypothesis that PCR exhibits better performance than antibody detection-based testing at early infection stages proves to be true, the progression of the test’s performances from the earlier study to the later study may also reflect earlier AH sampling in the present study than in the previous one. Increased levels of systematic AH sampling in cases of OT suspicion may explain this sampling being performed at an earlier stage of infection than had previously been the case, reflecting changes in the practice of the ophthalmologists.

In conclusion, our study data remind us that current biological diagnostic tools for OT must be used in combination in order to make the best of the precious ocular fluids sampled by ophthalmologists but also that the accuracy of the results also relies on the volume of the samples. This need for combining tests probably reflects the fact that clinical OT suspicion might occur at different disease stages, rendering parasites or antibodies more or less easily detected depending on the progression of the infection. To gain a better understanding of which parameters are relevant for the performances of the different diagnostic tests, prospective clinicobiological studies focused on correlating the performances of the different tests with clinical characteristics, such as the delay between symptom onset and ACP or the presence of a retinal scar, are critically needed. This would also be an opportunity to assess the performance of the clinical examination within the diagnostic procedure. Moreover, such studies might shed light on fundamental aspects of OT pathophysiology and pave the way to improvements in this infection’s diagnosis and treatment.

## MATERIALS AND METHODS

We included all the blood and ocular samples assessed from 1 January 2010 to 31 December 2018 in our laboratory for OT diagnosis without considering the strength of the clinical OT suspicion or the underlying clinical condition. All information was retrieved from our laboratory records. The samples originated from several medical centers, all situated in eastern France, including Strasbourg University hospital, Colmar hospital, Mulhouse hospital, Trévenans hospital, and Belfort hospital. Furthermore, two samples were sent by private clinical pathology laboratories and three directly by private practitioners.

Blood samples were employed for the serologic assays, which consisted of IgG and IgM titer determinations performed using the Liaison Toxo IgG and IgM assay (DiaSorin, Saluggia, Italy) (Se, 95.8%; Sp, 99.5%) (24) from January to March 2010, then starting from April 2010, the Architect Toxo IgG and IgM assay (Abbott, Rungis, France) (Se, 99.6%; Sp, 99.5%) (24). In cases of positive IgM titers, an avidity test was performed using an Architect Toxo IgG avidity kit (Abbott, Rungis, France) (Se, 89.3% at the acute phase and 87.1% at the latent phase) (25) to determine if the seropositivity corresponded to a primary infection or to a chronic infection. For the Architect Toxo IgG avidity kit, avidity was considered low for levels below 50%, high for levels above 60%, and doubtful for levels between those limits. Thus, a patient with positive IgM titers and an avidity result of below 50% was classified as presenting with a toxoplasmic primary infection.

Ocular samples were tested using three different techniques. Whenever possible, real-time quantitative PCR (qPCR) allowing the detection of the REP-529 sequence of T. gondii was performed (12). DNA extraction was carried out on the centrifugation pellet using a minimum of *5* μl of AH (12,000 rpm for 3 min) eluted in 100 μl of water by the use of a QIAamp DNA minikit (Qiagen, Courtaboeuf, France) until December 2017; then, after December 2017, the extraction procedure was carried out by the use of an automated MagNA Pure 96 nucleic acid extractor (Roche, Meyland, France). PCR was conducted using the Tox-9 and Tox-11 primers and Tox-HP-1 and Tox-HP-2 probes, as previously described (26). Each sample was tested four times in parallel, among which one of the tests contained an internal control for the presence of PCR inhibitors. In addition, each batch contained one positive control, one negative control, and a standardized scale. This protocol, along with external controls (i.e., evaluation by an external organization of the test performances in our laboratory), allowed performances of 100% Se and 100% Sp to be achieved at the threshold of 0.1 parasite/ml of liquid sample.

**FIG 3:**
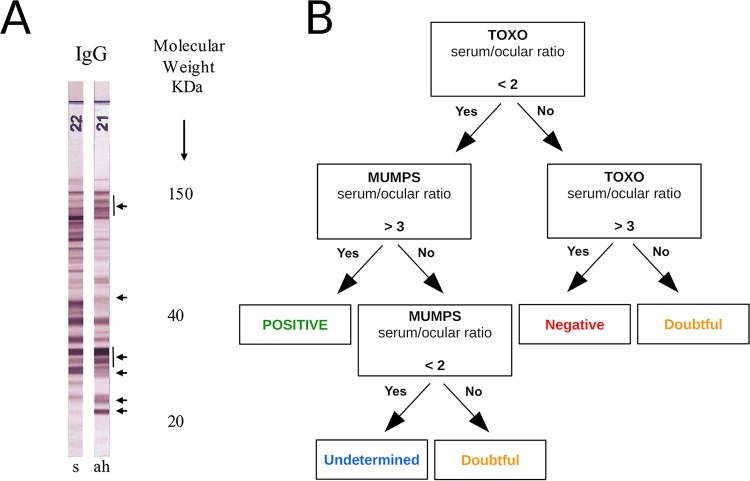
Serological assays in ocular samples. (A) Example of a positive test with different immune profiles in aqueous humor (ah) and serum (s). (B) Diagnostic algorithm using the Candolfi coefficient.

Whenever possible (e.g., if the remaining sample volume was sufficient), two serologic tests were conducted on each of these samples. We first performed an immunoblot assay comparing the IgG profiles of blood and ocular samples. To this end, a Toxoplasma Western blotting (WB) IgG/IgM (immunoprofile comparison) kit (Ldbio Diagnostics, Lyon, France) (Se, 62.8%; Sp, 92.8% [according to the manufacturer]) was employed. For an OT case, the toxoplasmic antigens detected in the ocular sample are expected to differ from those retrieved from the blood sample. This test thus remains reliable even if the blood-ocular barrier (BOB) is permeable to circulating antibodies. If immunoblotting carried out on the ocular samples demonstrated antigens that differed from those detected in the blood, the test result was considered positive (Fig. 3). This test was performed according to the manufacturer’s instructions by diluting a 25-μl AH sample or 10-μl blood serum sample in 1.2 ml buffer each.

Finally, the Candolfi coefficient (CC) was assessed by determination of antitoxoplasmic IgG titers in ocular and blood samples by the use of the enzyme-linked immunosorbent assay (ELISA)-IgG technique (12, 20). Thus, if the ratio of serum IgG to ocular IgG was <2, the “toxo” ratio was considered positive; if this ratio was >3, it was considered negative. The result was considered doubtful when the ratio was revealed to be between those two limits. The possibility of passive transudation of serum IgG into the eye through the BOB in cases of inflammation was assessed by comparing anti-mumps virus IgG titers in the serum and eye. If the ratio of serum IgG to ocular IgG was <2, the BOB was considered permeable to systemic antibodies; it was considered impermeable when >3 and doubtful when the value was between those limits. Thus, the CC was positive when the toxo ratio was <2 and the mumps ratio >3; the CC was negative when the toxo ratio was >3; the CC was considered doubtful when either the toxo or the mumps ratio was between 2 and 3; it was considered noninterpretable when the mumps ratio was <2 (Fig. 3). In any case, seropositivity for the mumps virus was required for the CC to be measured. The toxo ratio was assessed with a Platelia Toxo IgG kit (Bio-Rad, Marnes-La-Coquette, France), using a minimum of 20 μl AH sample diluted at 1/10 and a blood serum sample diluted at 1/300. The mumps ratio was assessed using a Captia mumps IgG kit (Trinity Biotech, Bray, Ireland) according to the instructions of the manufacturer.

Given the high specificity of each test performed (12), we considered OT to have been diagnosed correctly for every ocular sample that was positive for at least one of the previously described tests, including PCR, immunoblotting, and CC. This diagnosis algorithm has already been described in previously published reviews (12, 27).
