# Supplementary material for: Biological Diagnosis of Ocular Toxoplasmosis: a Nine-Year Retrospective Observational Study
Source: mSphere. 2019 Sep 25;4(5):e00636-19. doi: 10.1128/mSphere.00636-19 (PMC6763772; doi:10.1128/mSphere.00636-19)
Supplement: TABLE S1 [file mSphere.00636-19-st001.pdf]

Table S1 – Results of the tests performed for OT diagnosis on the 249 samples analyzed in Strasbourg University Hospital laboratory from 2010 to 2018.

N° case	Fluid	Serology	PCR	Candolfi coeff.	Immunoblot	OT
**1**	AH	**C**	N	U	I	N
**2**	AH	N	**P**	NR	NR	**P**
**3**	AH	**C**	N	**P**	**Di**	**P**
**4**	AH	N	N	N	NR	N
**5**	AH	N	N	U	I	N
**6**	AH	N	**P**	U	I	**P**
**7**	AH	**C**	N	D	I	N
**8**	AH	**A**	N	N	NR	N
**9**	AH	N	N	NR	NR	N
**10**	VH	**C**	**P**	U	**Di**	**P**
**11**	AH	**C**	**P**	D	**Di**	**P**
**12**	AH	N	N	NR	NR	N
**13**	AH	**C**	N	**P**	**Di**	**P**
**14**	AH	N	N	NR	NR	N
**15**	AH	NR	N	NR	NR	N
**16**	AH	N	N	NR	NR	N
**17**	AH	N	N	NR	NR	N
**18**	AH	NR	N	NR	NR	N
**19**	AH	N	N	NR	NR	N
**20**	AH	**C**	N	U	**Di**	**P**
**21**	AH	**C**	N	NR	N	N
**22**	AH	**C**	N	U	**Di**	**P**
**23**	AH	**C**	N	N	NR	N
**24**	AH	**C**	**P**	U	I	**P**
**25**	AH	NR	N	NR	NR	N
**26**	VH	NR	N	NR	NR	N
**27**	AH	**C**	N	D	N	N
**28**	AH	**C**	N	N	NR	N
**29**	AH	N	N	NR	NR	N
**30**	AH	**C**	N	U	I	N
**31**	AH	**C**	N	U	I	N
**32**	AH	NR	N	NR	NR	N
**33**	AH	N	N	NR	NR	N
**34**	AH	**C**	N	NR	NR	N

**35**	AH	**C**	N	U	I	N
**36**	AH	**C**	N	N	N	N
**37**	AH	**C**	N	D	NR	N
**38**	AH	N	NR	NR	NR	N
**39**	AH	**C**	N	N	I	N
**40**	AH	**C**	**P**	U	NR	**P**
**41**	AH	N	N	NR	NR	N
**42**	AH	**C**	N	NR	NR	N
**43**	AH	N	NR	NR	NR	N
**44**	AH	NR	N	NR	NR	N
**45**	AH	N	N	NR	NR	N
**46**	AH	**C**	N	NR	I	N
**47**	AH	N	N	NR	NR	N
**48**	AH	**C**	N	U	I	N
**49**	AH	NR	N	NR	NR	N
**50**	AH	**C**	N	U	I	N
**51**	AH	**C**	N	U	I	N
**52**	VH	N	N	NR	NR	N
**53**	AH	N	N	NR	NR	N
**54**	AH	NR	N	NR	NR	N
**55**	AH	N	N	NR	NR	N
**56**	AH	N	N	NR	NR	N
**57**	AH	N	N	NR	NR	N
**58**	AH	**C**	N	**P**	NR	**P**
**59**	AH	**C**	N	N	NR	N
**60**	AH	**C**	N	**P**	N	**P**
**61**	AH	**C**	N	N	NR	N
**62**	AH	**A**	N	U	I	N
**63**	AH	N	N	NR	NR	N
**64**	AH	N	N	NR	NR	N
**65**	AH	**C**	N	U	N	N
**66**	AH	**C**	**P**	**P**	I	**P**
**67**	AH	**A**	**P**	**P**	**Di**	**P**
**68**	AH	**C**	N	NR	I	N
**69**	AH	**C**	N	NR	IQ	N
**70**	AH	**C**	N	N	N	N
**71**	AH	**C**	N	N	N	N
**72**	AH	**C**	N	U	I	N
**73**	AH	NR	N	NR	NR	N

**74**	AH	N	N	NR	NR	N
**75**	AH	**C**	**P**	NR	I	**P**
**76**	AH	**C**	**P**	NR	I	**P**
**77**	AH	**C**	N	U	I	N
**78**	AH	N	N	NR	NR	N
**79**	AH	**C**	N	N	N	N
**80**	AH	**C**	N	NR	IQ	N
**81**	AH	**C**	N	NR	I	N
**82**	AH	**C**	**P**	NR	**Di**	**P**
**83**	AH	**C**	N	U	N	N
**84**	AH	**C**	N	NR	**Di**	**P**
**85**	AH	**C**	N	U	**Di**	**P**
**86**	AH	**C**	N	U	**Di**	**P**
**87**	AH	**C**	IQ	NR	I	N
**88**	AH	**C**	N	N	N	N
**89**	AH	**C**	N	U	I	N
**90**	AH	N	N	NR	NR	N
**91**	AH	**C**	**P**	NR	**Di**	**P**
**92**	AH	**C**	**P**	NR	NR	**P**
**93**	AH	**C**	**P**	U	I	**P**
**94**	AH	NR	N	NR	NR	N
**95**	AH	**C**	**P**	NR	**Di**	**P**
**96**	AH	NR	N	NR	NR	N
**97**	AH	N	N	NR	NR	N
**98**	AH	**A**	N	**P**	I	**P**
**99**	AH	**C**	**P**	NR	NR	**P**
**100**	AH	**C**	N	N	N	N
**101**	AH	N	NR	NR	NR	N
**102**	VH	**C**	N	N	N	N
**103**	VH	**C**	**P**	U	**Di**	**P**
**104**	AH	**C**	N	U	I	N
**105**	unknown	NR	**P**	NR	NR	**P**
**106**	AH	**C**	**P**	NR	**Di**	**P**
**107**	AH	**C**	N	NR	IQ	N
**108**	AH	**A**	**P**	NR	NR	**P**
**109**	AH	N	N	NR	NR	N
**110**	AH	NR	N	NR	NR	N
**111**	AH	N	N	NR	NR	N
**112**	AH	NR	N	NR	NR	N

**113**	AH	**C**	N	NR	N	N
**114**	AH	**C**	N	NR	**Di**	**P**
**115**	AH	**C**	N	NR	N	N
**116**	AH	N	N	NR	NR	N
**117**	AH	**C**	N	NR	NR	N
**118**	AH	N	N	NR	NR	N
**119**	AH	N	N	NR	NR	N
**120**	AH	**C**	N	NR	**Di**	**P**
**121**	AH	**C**	N	NR	N	N
**122**	AH	NR	**P**	NR	NR	**P**
**123**	AH	NR	N	U	I	N
**124**	AH	**C**	N	U	I	N
**125**	AH	N	N	NR	NR	N
**126**	AH	**C**	N	NR	NR	N
**127**	AH	**C**	N	N	N	N
**128**	AH	**C**	N	NR	I	N
**129**	AH	**C**	**P**	NR	I	**P**
**130**	AH	N	N	NR	NR	N
**131**	AH	**C**	N	U	I	N
**132**	AH	**C**	N	N	N	N
**133**	AH	N	N	NR	NR	N
**134**	AH	N	N	NR	NR	N
**135**	AH	N	NR	NR	NR	N
**136**	AH	N	N	NR	NR	N
**137**	AH	NR	N	N	I	N
**138**	AH	NR	N	N	N	N
**139**	AH	N	IQ	NR	NR	N
**140**	AH	N	N	NR	NR	N
**141**	VH	NR	N	NR	NR	N
**142**	AH	**C**	**P**	NR	I	**P**
**143**	AH	**C**	N	D	I	N
**144**	AH	**C**	N	D	I	N
**145**	AH	N	N	NR	NR	N
**146**	AH	**C**	N	NR	**Di**	**P**
**147**	AH	NR	N	NR	NR	N
**148**	AH	NR	N	NR	NR	N
**149**	AH	N	N	NR	NR	N
**150**	AH	N	N	NR	NR	N
**151**	AH	N	N	NR	NR	N

**152**	AH	**C**	**P**	NR	NR	**P**
**153**	AH	**C**	IQ	NR	I	N
**154**	AH	**C**	**P**	NR	NR	**P**
**155**	AH	**C**	**P**	NR	NR	**P**
**156**	AH	**C**	N	NR	NR	N
**157**	AH	NR	N	NR	NR	N
**158**	AH	N	N	NR	NR	N
**159**	AH	**C**	**P**	**P**	**Di**	**P**
**160**	AH	**C**	**P**	NR	NR	**P**
**161**	AH	**C**	N	**P**	I	**P**
**162**	AH	N	N	NR	NR	N
**163**	AH	**C**	N	NR	**Di**	**P**
**164**	AH	**C**	N	NR	IQ	N
**165**	AH	**C**	N	NR	NR	N
**166**	AH	**C**	N	NR	**Di**	**P**
**167**	AH	NR	N	NR	NR	N
**168**	AH	**C**	N	**P**	I	**P**
**169**	AH	N	N	NR	I	N
**170**	AH	NR	N	N	I	N
**171**	AH	**C**	N	NR	NR	N
**172**	AH	**C**	**P**	NR	N	**P**
**173**	AH	**C**	N	NR	**Di**	**P**
**174**	AH	**C**	N	NR	IQ	N
**175**	AH	**C**	N	NR	**Di**	**P**
**176**	AH	NR	N	NR	NR	N
**177**	AH	**C**	**P**	NR	I	**P**
**178**	AH	NR	N	NR	NR	N
**179**	AH	N	N	NR	NR	N
**180**	AH	N	N	NR	NR	N
**181**	AH	N	N	NR	NR	N
**182**	AH	**C**	N	N	N	N
**183**	AH	**C**	N	NR	I	N
**184**	AH	**C**	N	N	N	N
**185**	AH	**C**	N	NR	NR	N
**186**	AH	**C**	**P**	NR	NR	**P**
**187**	AH	**C**	N	D	**Di**	**P**
**188**	AH	**C**	N	NR	NR	N
**189**	AH	**C**	**P**	NR	NR	**P**
**190**	AH	**C**	N	**P**	**Di**	**P**

**191**	AH	N	N	NR	NR	N
**192**	AH	N	N	NR	NR	N
**193**	AH	**C**	N	**P**	**Di**	**P**
**194**	AH	**C**	N	N	N	N
**195**	AH	**A**	N	**P**	I	**P**
**196**	AH	**C**	N	U	I	N
**197**	AH	**C**	**P**	NR	NR	**P**
**198**	AH	**C**	N	NR	N	N
**199**	AH	**C**	**P**	NR	N	**P**
**200**	AH	**C**	N	NR	N	N
**201**	AH	N	N	NR	NR	N
**202**	AH	**C**	**P**	NR	NR	**P**
**203**	AH	NR	**P**	NR	NR	**P**
**204**	AH	NR	N	NR	NR	N
**205**	AH	**C**	N	U	I	N
**206**	VH	**A**	**P**	NR	**Di**	**P**
**207**	AH	NR	N	NR	NR	N
**208**	AH	**C**	**P**	NR	NR	**P**
**209**	AH	**C**	N	NR	**Di**	**P**
**210**	AH	**C**	**P**	NR	NR	**P**
**211**	AH	N	N	NR	NR	N
**212**	AH	**C**	N	NR	N	N
**213**	AH	**C**	**P**	NR	NR	**P**
**214**	AH	N	N	NR	NR	N
**215**	AH	**C**	**P**	NR	I	**P**
**216**	AH	NR	**P**	NR	NR	**P**
**217**	AH	**C**	N	NR	**Di**	**P**
**218**	AH	N	N	NR	NR	N
**219**	AH	NR	N	NR	NR	N
**220**	AH	**C**	N	NR	NR	N
**221**	AH	**C**	N	N	N	N
**222**	AH	**C**	**P**	**P**	**Di**	**P**
**223**	AH	D	N	N	N	N
**224**	VH	**C**	N	D	N	N
**225**	AH	**C**	N	N	N	N
**226**	AH	**C**	**P**	NR	NR	**P**
**227**	AH	**C**	N	U	**I**	N
**228**	AH	**C**	N	N	N	N
**229**	AH	**C**	N	**P**	I	**P**

**230**	AH	**C**	**P**	NR	NR	**P**
**231**	AH	**C**	N	U	I	N
**232**	AH	N	N	NR	NR	N
**233**	AH	NR	N	NR	NR	N
**234**	AH	**C**	**P**	NR	NR	**P**
**235**	AH	**C**	N	D	N	N
**236**	AH	**C**	N	NR	I	N
**237**	AH	**C**	N	NR	**Di**	**P**
**238**	AH	**C**	N	NR	**Di**	**P**
**239**	AH	**C**	**P**	NR	NR	**P**
**240**	AH	**C**	**P**	NR	NR	**P**
**241**	AH	**C**	**P**	NR	NR	**P**
**242**	AH	N	N	NR	NR	N
**243**	AH	**C**	N	D	I	N
**244**	AH	**C**	**P**	NR	NR	**P**
**245**	AH	N	N	NR	NR	N
**246**	AH	**C**	N	NR	N	N
**247**	AH	**C**	**P**	NR	**Di**	**P**
**248**	AH	**C**	N	NR	NR	N
**249**	AH	**C**	**P**	NR	NR	**P**

A: Acute; AH: Aqueous humour; C: Chronic; D: Doubtful; Di: Different; I: Identical; IQ: Insufficient quantity; N: Negative; NR: Not realized; P: Positive; U: Uninterpretable; VH: Vitreous humour;
